# Probing the bulk ionic conductivity by thin film hetero-epitaxial engineering

**DOI:** 10.1088/1468-6996/16/1/015001

**Published:** 2015-01-13

**Authors:** Daniele Pergolesi, Vladimir Roddatis, Emiliana Fabbri, Christof W Schneider, Thomas Lippert, Enrico Traversa, John A Kilner

**Affiliations:** 1 Paul Scherrer Institut, Department of General Energy Research, CH-5225, Villigen-PSI, Switzerland; 2 International Center for Materials Nanoarchitectonics (WPI-MANA), National Institute for Materials Science (NIMS), 1-1 Namiki, Tsukuba, Ibaraki 305-0044, Japan; 3 CIC Energigune, Albert Einstein 48, E-01510—Miñano (Álava), Spain; 4 Physical Science and Engineering Division, King Abdullah University of Science and Technology (KAUST), Thuwal 23955-6900, Saudi Arabia; 5 Department of Materials, Imperial College London, London SW7 2BP, UK

**Keywords:** pulsed laser deposition, high resolution transmission electron microscopy, oxygen ion conductors, impedance spectroscopy, ionic conductivity

## Abstract

Highly textured thin films with small grain boundary regions can be used as model systems to directly measure the bulk conductivity of oxygen ion conducting oxides. Ionic conducting thin films and epitaxial heterostructures are also widely used to probe the effect of strain on the oxygen ion migration in oxide materials. For the purpose of these investigations a good lattice matching between the film and the substrate is required to promote the ordered film growth. Moreover, the substrate should be a good electrical insulator at high temperature to allow a reliable electrical characterization of the deposited film. Here we report the fabrication of an epitaxial heterostructure made with a double buffer layer of BaZrO_3_ and SrTiO_3_ grown on MgO substrates that fulfills both requirements. Based on such template platform, highly ordered (001) epitaxially oriented thin films of 15% Sm-doped CeO_2_ and 8 mol% Y_2_O_3_ stabilized ZrO_2_ are grown. Bulk conductivities as well as activation energies are measured for both materials, confirming the success of the approach. The reported insulating template platform promises potential application also for the electrical characterization of other novel electrolyte materials that still need a thorough understanding of their ionic conductivity.

## 1. Introduction

The conducting properties of oxygen ion conducting oxides, such as Sm doped CeO_2_ (SDC), 8 mol% Y_2_O_3_ stabilized ZrO_2_ (YSZ), and La_(1−*x*)_Sr_*x*_Ga_(1−*y*)_Mg_*y*_O_(3−*δ*)_ (LSGM with typically *x* = *y* = 0.2), have been widely investigated for applications as electrolyte materials for solid oxide fuel cells (SOFCs) [1]. These are electrochemical devices that convert chemical energy from a fuel into electric energy with very high efficiency and small environmental impact. The importance these devices can have for the development of a power generation system to become more independent of fossil and nuclear fuel is widely recognized; however, the full exploitation of this technology is still limited by the very high operating temperatures required to achieve suitable ionic conductivity across the electrolyte [2]. The typical operating temperature of current state-of-the-art SOFCs is above 800 °C. Intense research activity is been focused on investigating the ionic conducting mechanism in oxide materials searching for the most suitable strategy to engineer materials with sufficiently high conductivity in the so-called intermediate temperature range, between 500 and 800 °C [3, 4].

In general, the micro-morphology of the conductor (average grain size, grain boundary, lattice distortions) can significantly affect the oxygen ion conduction [5]. In particular, grain boundaries appear often to block the oxygen ion migration. In the case of YSZ, the grain boundary shows conductivities up to two orders of magnitude lower than the bulk [6]. Other examples are Gd-doped CeO_2_ thin polycrystalline films which showed conductivity more than one order of magnitude lower than that of highly ordered films with small grain boundary regions [7]. This effect was attributed to the development of a negatively charged space charge layer surrounding the grain boundary core [8, 9].

Electrochemical impedance spectroscopy (EIS) measurements on polycrystalline ceramic pellets are typically used to investigate the total electrical conductivity of oxygen ion conducting oxides as a function of the temperature and the gaseous environment. Thus, the measured total conductivity is composed of different conduction pathways through the grain interior (bulk) and across the grain boundary. The separation of the bulk and grain boundary contributions from EIS impedance plots is routinely performed especially at relatively low temperatures [10]. By increasing the temperature (typically above 400 °C), both the grain and grain-boundary resistances decrease, and the frequency limitations of most of the commonly available instruments restrict the determination of the two contributions. At higher temperatures, only the availability of single crystals, sintered pellets with very large average grain size or highly ordered thin films allows a direct measurement the bulk oxygen ion conductivity. Single crystals are expensive and not always easily available and sintered pellets with micro-meter grain size can be difficult to fabricate. Therefore, highly ordered thin films, typically grown by pulsed laser deposition (PLD) on insulating substrates can be a valid alternative. However, it must be taken into account that in the case of in-plane electrical measurements of thin films the EIS plot always shows only one semi-circle independent on the sample morphology due to the parallel stray capacitance of the substrate. Thus, it is possible to assign the measured resistance to the grain interior conduction pathways only if the micro-structural analysis of the sample shows no evidence of significant grain separation (high angle grain boundaries).

By carefully tuning the deposition parameters, PLD allows the growth of thin films with well-defined microstructure and morphology from almost ideal single-crystalline samples to polycrystalline samples with nanometric average grain size. However, the fabrication and the electrical characterization of such model systems strongly depend on the availability of suitable deposition substrates. The substrate material should have good lattice matching with the film material to enable an ordered growth of the conductor, and it must be a good electrical insulator at high temperatures in order not to affect the in-plane electrical characterization.

Cubic perovskite single-crystalline wafers such as SrTiO_3_ (STO), YAlO_3_ (YAO) or LaAlO_3_ (LAO) are suitable substrates for a very well ordered growth of (doped-)ceria and LSGM. The latter has the same crystalline structure and a very similar lattice parameter to STO, allowing a cube-on-cube fully relaxed growth of the thin film [11, 12]. Differently, SDC has the cubic fluorite crystalline structure with lattice parameter *c* = 5.44 Å. A relatively good crystalline matching with STO can be achieved by way of the epitaxial symmetry (001)SDC//(001)STO and (100)SDC//(110)STO (i.e. with an in-plane 45° rotation of the SDC unit cell with respect to STO) [13]. In this case, SDC has an in-plane lattice mismatch of about −1.5%. In the case of YSZ, a cubic fluorite with lattice parameter of about 5.14 Å, the lattice mismatch with STO is much larger (about −7%) and the surface termination of the STO substrate seems to have a crucial role in tuning the epitaxial growth [14]. Nevertheless, highly ordered YSZ films were grown on (001)-oriented STO substrates [14–16]. Better interface quality might be achievable using (110)-oriented YAO substrates that allow a well-ordered epitaxial growth reducing significantly the lattice misfit [17].

However, it is well known that perovskite materials like STO are not good insulators at high temperatures [18]. As a consequence, the electrical conductivity of the substrate (much thicker than the film) can significantly affect the in-plane measurement of the film resistance [19].

Al_2_O_3_ substrates have very good insulating properties and are widely used for the fabrication of thin films of SDC and YSZ. The literature reports the growth of highly textured films of SDC on (0001)-oriented sapphire substrates [7], but also several examples of films showing multiple orientations on both (0001) and (1102)-oriented substrates [20–22]. YSZ thin films can be epitaxially grown on sapphire substrates, but these films usually show a columnar morphology consisting of parallel pillars (tens of nm in diameter) orthogonal to the substrate surface and extending through the entire film [16, 23]. Such morphology might be not appropriate for the in-plane investigation of the bulk conductivity since the conduction pathways across and along grain boundaries may dominate the conductivity.

Also MgO single-crystalline wafers offer suitable insulating properties, and indeed YSZ thin-films with very good crystalline quality have been grown on this substrate [15, 17]. However, the lattice mismatch at the interface with SDC and YSZ is as large as about 20% leading often to polycrystalline microstructures [13] and to highly defective film-substrate interfaces which may also affect in-plane conductivity measurements providing a fast ion conduction pathways along dislocation lines [15].

The problem of combining a good crystallographic matching between film and substrate, and good insulating properties of the deposition substrate has been successfully addressed in [13] and [24]. In these studies (001)-oriented MgO single-crystalline wafers were used as insulating substrates and a thin film of STO grown *in situ* on the substrate surface provided the suitable seed layer allowing the highly ordered growth of the conductor. With this design the electrical conductivity of the thin STO buffer layer, about 10 nm thick, was negligibly small compared to that of the ionic conducting films under investigation.

Both STO and MgO have a cubic symmetry but different crystalline structures, i.e. perovskite and rock salt, respectively. This in our experience often results in a sample fabrication with a limited yield probably due to small deviations of the deposition parameters that are difficult to control and typically lead to a columnar growth.

To circumvent this problem and to allow a better yield for the reproducible growth of a STO seed layer on MgO without the presence of high angle grain boundaries, a new template platform for the growth of highly textured ionic conducting thin films has been engineered. The first layer of the new template platform is BaZrO_3_ (BZO), a cubic perovskite with a lattice parameter of about 4.19 Å, very similar to the lattice parameter of MgO (4.21 Å). The literature reports several examples of a bi-axially textured growth of doped [25, 26] and undoped [27, 28] BZO on MgO and very similar results were obtained for this work. The second layer of the template platform is STO according to the MgO + BZO + STO scheme.

## 2. Experimental details

A custom made PLD system (AOV Ltd) equipped with load-lock chamber and high-pressure reflection high energy electron diffraction (HP-RHEED) was used for the fabrication of the samples. Sintered ceramic pellets of BaZrO_3_, SrTiO_3_, CeO_2_, Ce_0.75_Sm_0.15_O_2−*δ*_ and 8 mol% Y_2_O_3_ stabilized ZrO_2_ were prepared in our laboratory and used as targets for PLD. Commercially available (100) oriented MgO single-crystalline wafers were used as deposition substrates. The substrates were ultrasonically cleaned in acetone and isopropanol before loading into the vacuum chamber. A resistive heater allowed setting the temperature of the deposition substrate up to the desired value. The thermal contact between the substrate and the heater was provided by platinum paste. The same deposition parameters were used for all materials changing only the number of laser pulses in order to grow films of the desired thickness. The temperature of the heater during the deposition was kept at 800 °C. The thermal contact between heating plate and substrates was provided by Pt paste. In this experimental condition the temperature of the substrate was about 720 °C, estimated by previous calibration with a thermocouple in good thermal contact with the heating plate.

The target-to-substrate distance was set to 65 mm. The vacuum chamber was evacuated down to the base pressure of about 10^−6^ Pa and then a high purity oxygen partial pressure of 5 Pa to vacuum was used during the film growth.

A KrF excimer laser (Coherent Lambda Physik GmbH) with a wavelength of 248 nm and a pulse width of 25 ns was focused on the target material in a spot area of about 4 mm^2^ with an energy of about 80 mJ (measured at the target surface). A repetition rate between 2 and 5 Hz was used. During the ablation process, each target was rotated and oscillated, for a uniform ablation of the target surface. A stainless steel shield avoided cross-contamination of the targets. A programmable control unit allowed programming multistep deposition processes selecting for each subsequent step the target material, the target oscillation amplitude and velocity, the number of laser shots and the laser frequency for each target.

X-ray diffraction (XRD) (PANalytical X’pert Pro MPD, *λ* = 0.1540 nm) analysis was used for the calibration of the deposition rate by x-ray reflectivity (XRR) and to investigate the crystalline structure of the films. The growth mechanism of the thin films was monitored *in situ* by HP-RHEED.

High-resolution transmission electron microscopy (HR-TEM) was carried out using a Tecnai F20ST electron microscope equipped with a high-angle annular dark-field detector and operated at 200 kV. Samples for HR-TEM analysis were prepared by standard techniques, including mechanical polishing followed by ion milling using a Fischione 1010 ion mill.

For the electrical measurements, two parallel Pt electrodes about 100 nm thick were fabricated on the film surface by electron beam deposition at room temperature. 5 nm thick Ti layers were used to improve Pt adhesion. EIS measurements were performed between 100 mHz and 1 MHz in air, varying the temperature between 400 and 700 °C, using a multichannel potentiostat VMP3 (Bio-Logic).

## 3. Results and discussion

**Figure 1.**
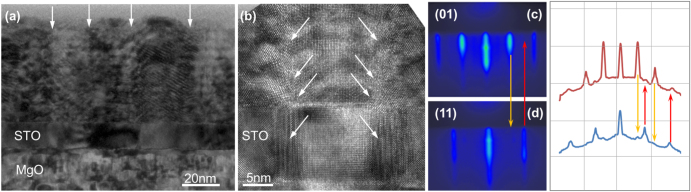
(a) TEM cross section micrograph of a CeO_2_/YSZ multilayer grown on STO-buffered MgO showing the columnar morphology originating at the STO buffer layer. HR-TEM image at high magnification of the same sample is shown in (b). The arrows show the grain boundary regions. The heterostructure is epitaxially oriented with the substrate but shows two different in-plane orientations 45° tilted one another, as evidenced in (c) and (d): RHEED patterns and intensity profiles acquired after the growth of the STO layer along the (01) crystallographic direction of the substrate (c) and along the (11) direction (d).

To directly measure the bulk conductivity of ionic conducting oxides, thin film with high crystalline quality grown on insulating substrates can be used. Good results have been achieved for thin films of doped ceria and for CeO_2_/YSZ multilayered heterostructures using STO-buffered MgO substrates [13, 24]. However, in our experience, this strategy has a limited reproducibility and tiny variation of the deposition conditions (difficult to control) and/or of the quality of the substrate surface, often result in a columnar morphology, epitaxially oriented with the substrate but with large grain boundary regions. As a consequence, the fabricated samples require an individual HR-TEM analysis to select those with no evidence of high angle grain boundary and columnar morphology. An example can be found in figure 1(a) that shows the cross section HR-TEM image of a multilayered heterostructure made of 15 CeO_2_/YSZ bilayers grown epitaxially oriented with the STO-buffered MgO substrate.

The entire heterostructure is (001) epitaxially oriented with the substrate but has a clear columnar morphology. Such a columnar morphology, highlighted with the arrows in figure 1(a), is originated at the STO buffer layer that shows grains with the expected in-plane epitaxial orientation, i.e. (100)STO//(100)MgO, alternated with grains 45° tilted in-plane, i.e. (110)STO//(100)MgO. For these grains, identifiable only by HR-TEM, there cannot be any crystallographic matching at the interface between STO and MgO, thus the origin of the driving force for the nucleation of these tilted grains is not clear.

In some cases it was even possible to diagnose *in situ* the formation of in-plane tilted grains during the growth of the STO buffer layer. Figures 1(c) and (d) show the RHEED patterns acquired after the growth of the STO buffer layer on MgO along the (01) and (11) in-plane directions, respectively. The analysis of the distribution of the intensity along the images shows that the in-plane (11) RHEED reflections are present also when the electron beam was aligned towards the (01) crystalline direction, and vice versa. In the case of an (almost ideal) ordered bi-axially textured growth, no overlap of the two RHEED patterns has to be expected.

To circumvent this problem, an additional seed layer of BZO was deposited between the MgO substrate and the STO buffer layer according to the design: MgO + BZO + STO.

**Figure 2.**
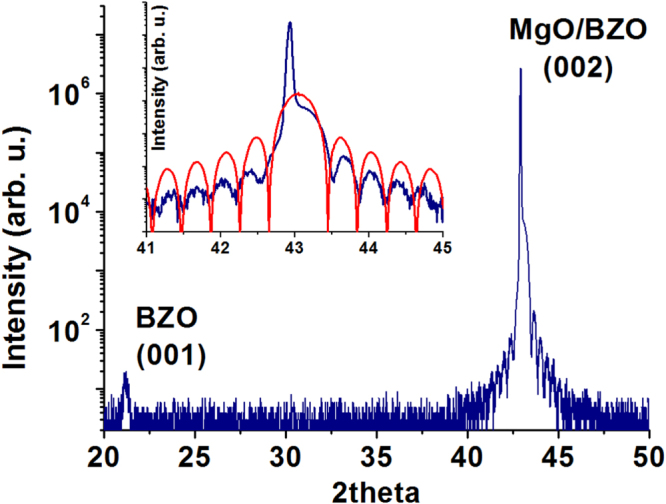
2*θ*/*θ* scan plot of a 250 Å thick film of BZO on MgO. The inset shows a magnification of the angular region around the (002) peak and the fit (red line) of the interference fringes calculated for a film thickness of 57 unit cells.

Figure 2 shows the XRD plot of a BZO film grown on MgO. The film is about 250 Å thick, as measured by XRR. Size effect interference fringes are visible around the (002) reflection line of BZO/MgO indicating a good interface quality. The relative spacing between the interference fringes is consistent with a film thickness of about 57 unit cells (≈239 Å), in good agreement with the XRR estimation of the thickness.

BZO does not reduce significantly the large lattice mismatch between MgO and STO but the formation of the previously described 45° in-plane tilted grains was never observed when the additional BZO layer was used. We believe that the different crystalline structure, cubic perovskite for BZO instead of rock salt for MgO, could be the reason that allows a more ordered and reproducible growth of the STO seed layer.

**Figure 3.**
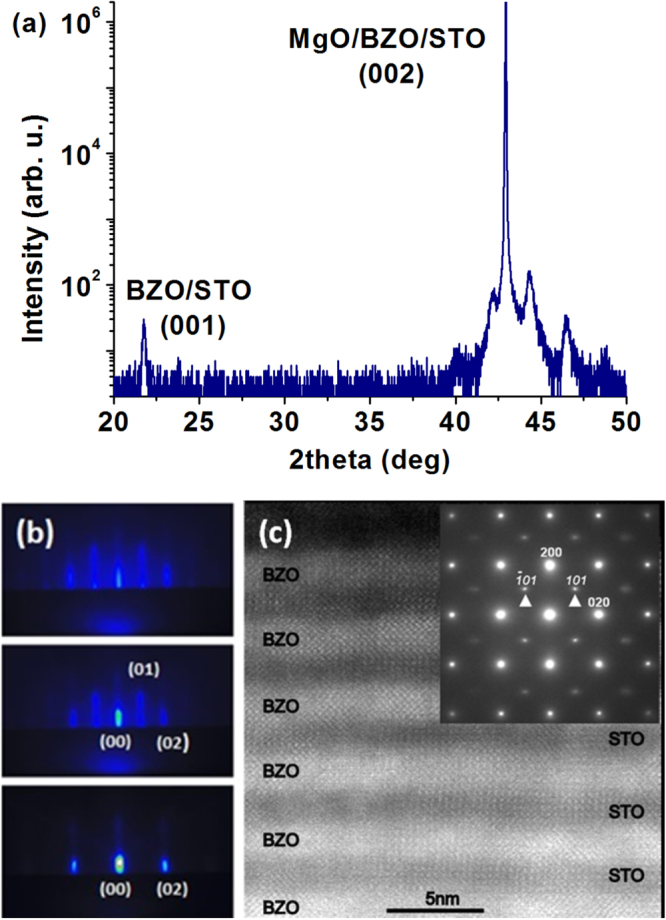
MgO + BZO + (STO + BZO) × 20 superlattice. (a) The XRD analysis shows the epitaxial orientation and the satellite peaks of the superlattice. (b) The RHEED patterns (from bottom to top MgO → BZO → STO) reveal an almost ideal layer-by-layer growth of the whole heterostructure. (c) The HR-STEM analysis shows a highly ordered growth of the complete structure with a very small degree of out-of-plane misalignment, as revealed by the SAED pattern shown in the inset. Spots from the MgO substrate are marked in bold while weak spots marked with arrowheads stem from the BZO/STO heterostructure.

The growth and the electrical properties of the MgO + BZO + STO template platform were studied using a multi-layered heterostructure of the two materials. Twenty STO/BZO bilayers were grown on BZO-buffered MgO according to the scheme: MgO + BZO + (STO + BZO) × 20. The thickness of each layer of BZO and STO was about 2 nm. Figure 3 shows the XRD, RHEED and HR-TEM analysis of this sample.

The BZO/STO heterostructure has a very high crystalline quality with no evidence of grain separation and a very small degree of out-of-plane misalignment as revealed by selected area electron diffraction (SAED) patterns. RHEED showed streaky patterns suggesting a Frank–van der Merwe (layer-by-layer) growth mechanism. The RHEED patterns did not show any significant change after the deposition of the first BZO film indicating that a well-oriented perovskite structure was preserved up to the 21st layer of the heterostructure. However, Fourier-transformed HR-TEM images revealed in some parts of the heterostructure a precession of the [100] direction of STO or BZO within a range of 4–5° and the average linear spacing between low angle grain boundary at the interface with the substrate was about 5 nm. A detailed characterization of the interfaces of BZO/STO heterostructures is also reported in reference [27] and the growth of BZO/STO superlattices on STO substrates is reported in reference [28]. The microstructural properties of the BZO/STO double buffer layer were investigated to promote the ordered growth of YBa_2_Cu_3_O_7−*δ*_ films on MgO and to avoid chemical interaction at the MgO/(Ca,Ba)Nb_2_O_6_ interface [27]. Antiphase boundaries and dislocations were found as lattice defects along the BZO layer, while the majority defects at the STO/BZO interface were identified as misfit dislocations introduced to release the excess strain. It was also found that these dislocations contribute to terminate the propagation of the BZO lattice defects toward the STO layer. This effect leads to a high crystalline quality of the STO surface [27].

The BZO/STO multi-layered heterostructure described in figure 3 was used to check the insulating properties of the BZO/STO double buffer layer used for the fabrication of the template platform, as will be discussed later.

**Figure 4.**
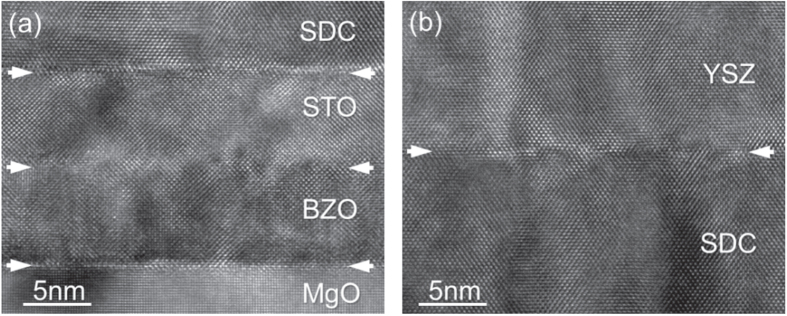
HR-TEM image of an SDC film grown on the MgO + BZO + STO template platform (a). The SDC layer can be used for the highly ordered growth of YSZ thin films (b).

**Figure 5.**
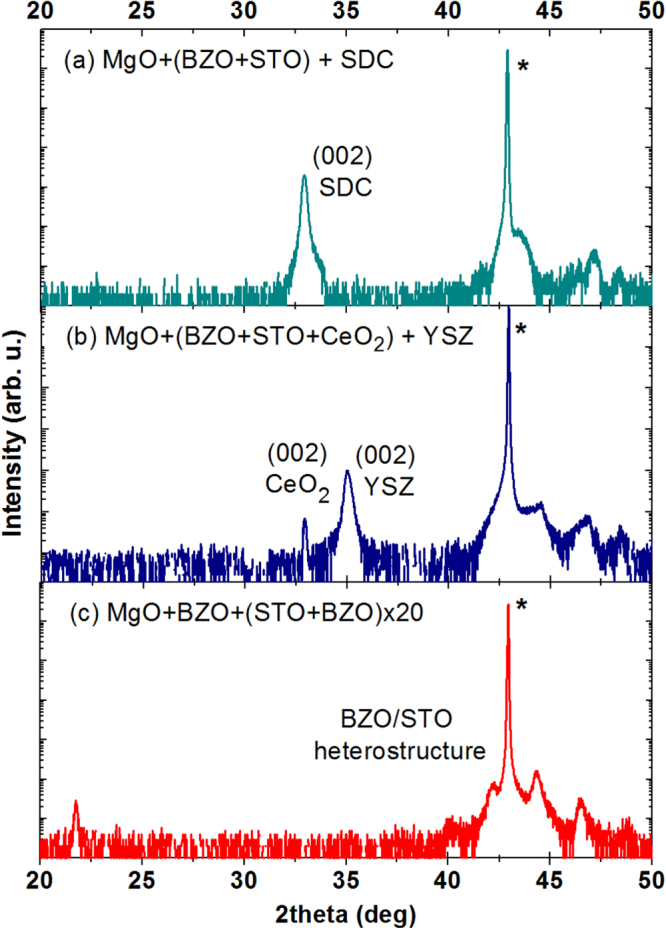
XRD analysis of the SDC (a) and YSZ (b) films grown on the MgO + BZO + STO template platform. For the YSZ film an additional thin layer of CeO_2_ was used. For comparison the XRD plot of the BZO/STO heterostructure is reported (c). The asterisk indicates the (002) reflection of the MgO substrate.

Highly ordered SDC films and SDC/YSZ heterostructures were grown on the MgO + BZO + STO template platform, as demonstrated by figure 4(a) showing the HR-TEM cross section image of an SDC/YSZ heterostructure in the region of the double buffer layer, and by figure 5(a) showing the XRD analysis of one of the SDC films, about 200 nm thick, used for the EIS characterization. The thicknesses of the BZO and STO buffer layers of the template platform used for the electrical characterizations were about 5 nm.

From the angular position of the (002) diffraction peak of the SDC film a value of about 5.443 Å can be estimated for the out-of-plane lattice parameter revealing a relaxed crystalline structure as expected for a 200 nm thick film.

In the case of YSZ, it was not possible to reproduce the film quality reported in [15] and [16] for YSZ films grown on STO single-crystalline substrates. The growth on the STO surface of the template platform resulted in thin films mostly (001)-epitaxially oriented but with evidence of (111) minor orientation. This could be expected due to the larger lattice mismatch between YSZ film and STO substrate with respect to SDC, which may explain the different results obtained with the two materials. Moreover, the literature reports that orientation and morphology of YSZ thin films grown on STO single-crystalline wafers strongly depend on the surface termination of the substrate. In reference [14] for example, epitaxially oriented and highly ordered YSZ films could be obtained only on SrO-terminated surfaces. It might be possible to attain a SrO-terminated surface by depositing an ultra-thin layer of SrO on the STO buffer layer but this approach requires atomically flat surfaces that were not obtained for our STO films. Alternatively, a thin layer of doped or undoped CeO_2_ can be used to promote a bi-axially textured growth of the YSZ film free from high angle grain boundary as shown in [24]. As an example, figure 4(b) shows the HR-TEM image of the YSZ film grown on the SDC film shown in figure 4(a).

Following this second approach, a thin layer of undoped CeO_2_, about 5 nm thick, was grown *in situ* on the template platform before the deposition of the YSZ films used for the electrical characterization. These YSZ films were about 200 nm thick and showed an epitaxial orientation with the template platform as revealed by the XRD analysis reported in figure 5(b). The out-of-plane lattice parameter measured for the YSZ film is about 5.136 Å consistent with a relaxed crystalline structure. Also in this case the thicknesses of the BZO and STO buffer layers of the template platform were about 5 nm.

For comparison and for a clear identification of the small features visible on the right side of the peak of the substrate, figure 5(c) shows the XRD plot of the BZO/STO heterostructure described above.

**Figure 6.**
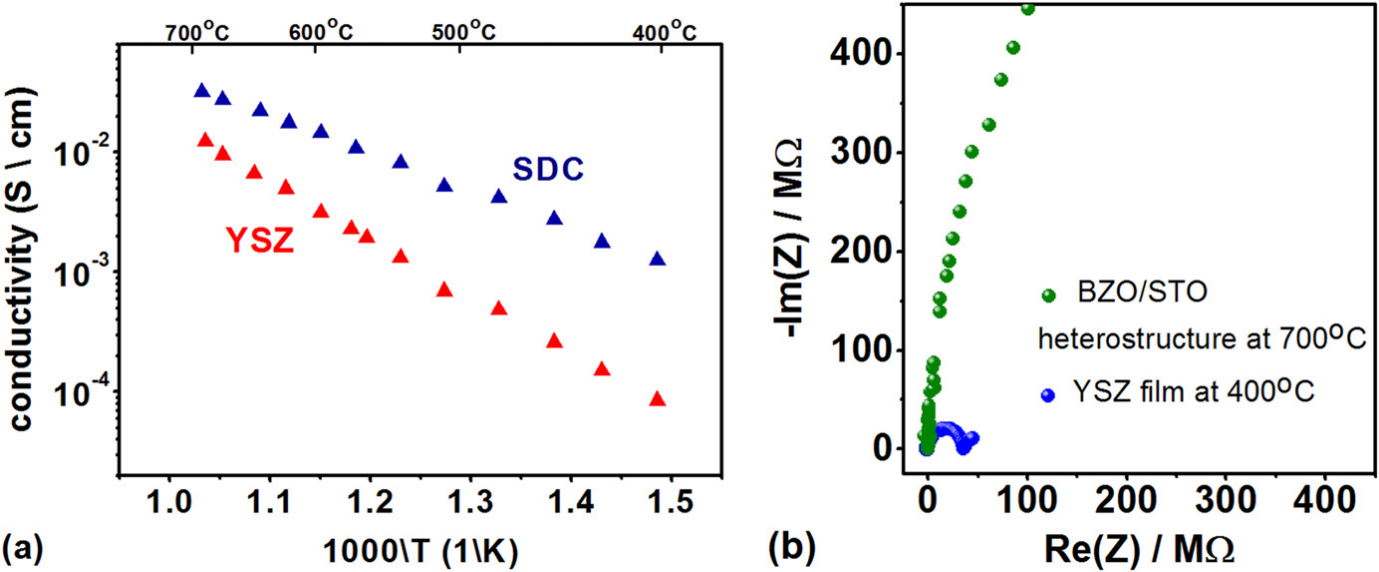
(a) Temperature dependence of the total electrical conductivity of SDC and YSZ thin films grown on the MgO + BZO + STO template platform. The calculated activation energies are 0.69 and 1.02 eV for SDC and YSZ, respectively. (b) Comparison of the Nyquist plots of the YSZ film at the lowest measured temperature (the larger value of resistance) and the BZO/STO superlattice (20 bilayers) at the highest temperature (the smaller value of resistance).

Figure 6(a) shows the in-plane electrical conductivities of the SDC and YSZ films that were measured by EIS in the temperature range between 400 and 700 °C in air using two Pt electrodes at a distance of about 1 mm deposited onto the surface of the films. The SDC film showed activation energy of about 0.69 eV while for the YSZ film an activation energy of about 1.02 eV was measured.

These values together with the measured conductivities are in very good agreement with the value of bulk conductivities and activation energies reported in the literature for the two materials. In particular, the measured conductivity of the SDC film matches very well those measured for almost ideal single-crystalline 10% Gd-doped CeO_2_ films grown (111)-oriented on sapphire [7] and for (001)-oriented SDC films grown on STO-buffered MgO [12]. Concerning YSZ, the measured conductivity and activation energy are in very good agreement with values measured for 9.5 mol% YSZ single crystals [29].

It is worth highlighting here, that using sintered ceramic pellets with a typical average grain size in the range of few hundreds of nm, the EIS measurements allow to discriminate the bulk conductivity very effectively but often, depending on sample fabrication and morphology, only in the temperature range below ∼500 °C. An example can be found in [30] where the bulk conductivity of yttria doped ceria was measured using single crystals prepared by inductive melting and using sintered ceramic pellets of the same material. A very good agreement was found between the two methods up to a maximum temperature of about 500 °C. However, in the temperature range of interest for practical applications, i.e. between 500 and 800 °C, only a sample morphology characterized by large average grain size and small grain boundary regions allows the direct measurement of the conducting properties of the grain interior. Such a morphology can be difficult to achieve with polycrystalline sintered samples, and highly ordered thin films grown on insulating substrates can be a valid alternative. Moreover, the described template platform could be a suitable substrate for the highly ordered and grain-boundary-free growth of ionic conducting thin films or multi-layered heterostructures used to investigate the effect of the lattice distortion on the ionic conductivity [31].

Concerning the contribution to the measured conductivity of the BZO/STO double buffer layer, figure 6(b) shows the comparison of the EIS plots measured for the YSZ film at the lowest temperature of 400 °C (the largest resistance) and for the heterostructure MgO + BZO + (STO+BZO) × 20 made with twenty STO/BZO bilayers at the highest temperature of 700 °C (the smallest resistance). The Pt electrodes were patterned with the same geometry on both samples allowing a direct comparison of the measured resistances. Taking into account that for the electrical characterizations of the SDC and YSZ films only one BZO/STO bilayer was used, these measurements clearly show that the electrical conductivity of the template platform can be neglected. Unfortunately, due to limitations of the EIS setup used for this study (in particular the geometry and dimension of the electrodes) the large electrical resistance of the BZO/STO bilayer, as well as of the heterostructure consisting on 20 BZO/STO bilayers, precluded the accurate conductivity measurement in the investigated temperature range. The use of interdigitated micro-electrodes or micropatterned side-electrodes as those reported in reference [32] has to be considered for potential future applications of this template platform; As an example for the investigation of the strain-related effects on the ionic conductivity using ionic conducting multilayered structures or single layers whose thickness is in the range of few (tens) nm.

Finally, the measured values of conductivities and activation energies strongly suggest that surface and/or interface effects (segregation, depletion, strain), whose investigation is beyond of the scope of the present study, are not noticeable and the conductivity of the relaxed bulk volume dominates the transport properties as expected for film as thick as 200 nm. Moreover, as previously observed with similar samples [24], EIS measurements showed no evidence of chemical interdiffusion between adjacent layers.

## 4. Conclusions

An electrically insulating template platform allowing a bi-axially textured grain-boundary-free growth of SDC and YSZ thin films along the (001) crystallographic orientation was fabricated by PLD. The template platform consists of an epitaxial thin film heterostructure made of a double buffer layer of BZO and STO grown on MgO single-crystalline substrates. The complete growth of the template platform and of the ionic conductors was performed *in situ* and analyzed by RHEED, XRD and HR-TEM. The introduction of the BZO layer allowed a more reproducible growth of the STO seed layer that provides the suitable crystallographic matching for the ordered growth of the conductor. It is worth pointing out, that the overall crystalline quality of the fabricated samples in terms of the presence of local defects and interface roughness was found to be very similar to values reported in the literature for similar heterostructures [24]. EIS characterization showed that the presence of these local defects does not affect the conductivity measurements.

EIS analysis showed that the electrical conductivity of the template platform can be considered negligible compared to the conductivity of the ionic conducting thin films. In other words, the use of such platform provides a distinct advantage because it allows studying the ionic conductivity of highly ordered thin films not affected by the electrical conductivity of the substrate. In fact, the two ionic conducting thin films of SDC and YSZ showed the typical bulk conductivities and activation energies of the two materials.

This template platform is expected to allow the highly ordered growth and the direct measurement of the bulk conducting properties of other oxygen-ion conductors like LSGM (cubic perovskite with lattice parameter of about 391 Å), perovskite Li-ion conductors like lithium lanthanum titanate (LLTO) with lattice parameter between 3.87 and 3.88 Å, and the new family of oxygen ion conductors based on the ferroelectric perovskite Na_0.5_Bi_0.5_TiO_3_ [33] whose pseudocubic unit cell possesses a lattice parameter of about 3.88 Å. Moreover, the same template platform can be used for the fabrication of highly ordered multi-layered heterostructures used to investigate the effects of the lattice distortions on the ionic conductivity [24, 31, 34].
